# Effects of chronic HIV-1 Tat exposure in the CNS: heightened vulnerability of males versus females to changes in cell numbers, synaptic integrity, and behavior

**DOI:** 10.1007/s00429-013-0676-6

**Published:** 2013-12-19

**Authors:** Yun Kyung Hahn, Elizabeth M. Podhaizer, Sean P. Farris, Michael F. Miles, Kurt F. Hauser, Pamela E. Knapp

**Affiliations:** 1 Department of Anatomy and Neurobiology, Virginia Commonwealth University, Medical College of Virginia (MCV) Campus, PO Box 980709, Richmond, VA 23298-0709 USA; 2 Department of Pharmacology and Toxicology, Virginia Commonwealth University, MCV Campus, Richmond, VA 23298-0613 USA; 3 Institute for Drug and Alcohol Studies, Virginia Commonwealth University, Richmond, VA 23298 USA

**Keywords:** Striatum, NeuroAIDS, Tat, HIV-1, Sex, Behavior

## Abstract

HIV-associated damage to the central nervous system results in cognitive and motor deficits. Anti-retroviral therapies reduce the severity of symptoms, yet the proportion of patients affected has remained the same or increased. Although approximately half of HIV-infected patients worldwide are women, the question of whether biological sex influences outcomes of HIV infection has received little attention. We explored this question for both behavioral and cellular/morphologic endpoints, using a transgenic mouse that inducibly expresses HIV-1 Tat in the brain. After 3 months of HIV-1 Tat exposure, both sexes showed similar reduced open field ambulation. Male Tat^+^ mice also showed reduced forelimb grip strength and enhanced anxiety in a light–dark box assay. Tat^+^ males did not improve over 12 weeks of repeated rotarod testing, indicating a motor memory deficit. Male mice also had more cellular deficits in the striatum. Neither sex showed a change in volume or total neuron numbers. Both had equally reduced oligodendroglial populations and equivalent microglial increases. However, astrogliosis and microglial nitrosative stress were higher in males. Dendrites on medium spiny neurons in male Tat^+^ mice had fewer spines, and levels of excitatory and inhibitory pre- and post-synaptic proteins were disrupted. Our results predict sex as a determinant of HIV effects in brain. Increased behavioral deficits in males correlated with glial activation and synaptic damage, both of which are implicated in cognitive/motor impairments in patients. Tat produced by residually infected cells despite antiretroviral therapy may be an important determinant of the synaptodendritic instability and behavioral deficits accompanying chronic infection.

**Electronic supplementary material:**

The online version of this article (doi:10.1007/s00429-013-0676-6) contains supplementary material, which is available to authorized users.

## Introduction

Combination antiretroviral therapy (cART) has caused a dramatic decline in human immunodeficiency virus (HIV)-associated dementia and mortality. However, the overall prevalence of more moderate motor and cognitive deficits, collectively termed HIV-associated neurocognitive disorders (HAND), has remained similar in cART treated patients (Robertson et al. 2007; McArthur et al. 2010; Heaton et al. 2011). In resource-limited countries, where estimates are that only 36 % of the HIV-positive population receives cART, the severity and/or prevalence of neurocognitive disorders is higher (Nakasujja et al. 2005; Wong et al. 2007; Joint United Nations Programme on HIV/AIDS 2010a; Heaton et al. 2010; Tozzi et al. 2005). HAND occurs even in aviremic, cART-treated individuals, suggesting that many anti-retroviral regimens fail to reverse neurological damage, even while lengthening survival (Heaton et al. 2010, 2011; McArthur et al. 2010).

Approximately half of the 40 million persons infected with HIV-1 worldwide are women (Joint United Nations Programme on HIV/AIDS 2010a). The incidence of HIV is higher in men compared with women in all ethnic groups in the US (Joint United Nations Programme on HIV/AIDS 2010b; Centers for Disease Control and Prevention 2009) and Europe (European Centre for Disease Prevention and Control/WHO Regional Office for Europe 2011), and most studies on HAND epidemiology in western countries focus on males. Studies both pre- and post-cART have reported no difference in rate of HIV-associated neurocognitive complications between sexes in the United States (Robertson et al. 2004; Bouwman et al. 1998). In other studies, the pre- and post-cART risk of similar complications in both developed and resource-limited regions was reportedly higher in females (Wojna et al. 2006; Chiesi et al. 1996; Gupta et al. 2007; Hestad et al. 2012). In contrast, Joska (Joska et al. 2011) found the male genotype to be associated with HIV-related cognitive dysfunction, and Liu (Liu et al. 1996) reported that male HIV^+^ injection drug abusers had higher cognitive impairment rates. A recent survey of HIV status among patients in the National Epidemiological Survey on Alcohol and Related Conditions found HIV^+^ males but not HIV^+^ females had higher rates of mood, anxiety, and personality disorders than their same-sex HIV^−^ counterparts, even when adjusted for socio-demographic factors (Lopes et al. 2012). Disparities between the sexes in health care services and access to HIV treatment may be confounds, as globally, HIV-infected women are more likely to live in poverty, and have lower literacy levels, higher injection drug abuse rates, and poorer mental health. These considerations might significantly influence either CNS vulnerability to HIV, or a diagnosis of neurocognitive disability (Basso and Bornstein 2000; Farinpour et al. 2003; Maki and Martin-Thormeyer 2009).

We utilized a mouse model with conditional expression of HIV-1 Tat_1–86_ in the CNS to test whether biological sex can influence HIV-related motor and cognitive outcomes. Intriguingly, males showed more impairment in motor, learning, and anxiety tests. Since multiple brain regions are undoubtedly involved in such varied deficits, we specifically examined striatum, which is clearly impacted by HIV and is also involved as a coordinating center in multiple motor, memory, and motivational/emotional tasks and behaviors. At a cellular level, Tat induction significantly altered non-neuronal populations in both sexes. Importantly, males had greater increases in astroglia and activated microglia, and increased TUNEL^+^ neurons. Dendritic spine density was decreased more significantly in Tat^+^ males, and both inhibitory and excitatory pre- and post-synaptic proteins were more significantly altered.

The substantial changes in synaptic organization observed in male mice after chronic HIV-1 Tat exposure may make males more vulnerable to motor and social/cognitive behavioral impairment. We propose that HIV-1 Tat differentially affects aspects of inflammation and glial reactivity/remodeling in males and females and that these factors may underlie sex differences in synaptic reorganization and behavioral impairment in Tat transgenic mice, and perhaps also in neuroAIDS patients.

## Materials and methods

Animal studies were approved by the Institutional Animal Care and Use Committee at Virginia Commonwealth University.

### Animals

Doxycycline (DOX)-inducible HIV-1_IIIB_ Tat_1–86_ transgenic mice were generated as described (Hauser et al. 2009; Fitting et al. 2010b, 2013; Bruce-Keller et al. 2008). Since *tat* transgene activity is controlled by the glial fibrillary acidic protein (GFAP) promoter, Tat protein expression in the CNS is limited to astroglia. All mice were genotyped to confirm the presence of *tat* and *rtTA* transgenes. Chronic CNS Tat expression was induced by feeding chow containing DOX (Harlan Laboratories, Inc., Indianapolis, IN; 6 g/kg) to mice starting at 3 months of age for a 12-week period. Control Tat^−^ mice were also fed DOX-chow to control for off-target drug effects. In some behavior studies (grip strength, open field test, light/dark box test), other mice of the same age and genotype received normal chow through the experimental period so that data could be normalized. All mice were fed ad libitum. Mice were habituated to the study room for 60 min before all behavior tests. GFAP transcription is sensitive to levels of many hormones and growth factors, yet basal levels of GFAP transcription using such promoters were reported to be roughly similar in males and females in several studies (Cordeau et al. 2008; Cho et al. 2009; Lundkvist et al. 1999). Doxycycline pharmacokinetics in males and females are also similar (Binh et al. 2009).

### Rotarod assay

An accelerating rotarod assay was used to evaluate motor performance/coordination and motor memory (Shiotsuki et al. 2010; Brooks and Dunnett 2009; Liu et al. 2010; Jones and Roberts 1968), which are adversely affected in HAND (Tozzi et al. 2005; Goodkin et al. 1997; Simioni et al. 2010). All mice were naïve to the apparatus until the first test day. The Rotamex-5 treadmill (Columbus Instruments, Columbus, OH) consists of a rubber-surfaced, 3.0-cm diameter cylindrical treadmill connected to a computer-controlled stepper motor-driven drum. For each trial, mice were placed in individual compartments; the rod was gradually accelerated from 1 to 40 rpm, in 1 rpm increases per 15 s. Acceleration continued until 40 rpm was reached or the animal fell from the rod. Falls were detected by sensors at the bottom of each compartment. Rotarod testing began after 1 week DOX treatment, and was performed once per week for 4 weeks (13–16 weeks of age). After a 4-week break, testing was resumed during the third month of DOX treatment (21–24 weeks of age).

### Forelimb grip strength test

Forelimb grip strength was measured as a second indication of potential motor deficits (Chatillon^®^ DFE II grip strength meter, Ametek Test and Calibration Instruments, Largo, FL). Mice were held near the tail base and lowered toward the apparatus until the bar was gripped firmly with both forepaws. The mouse was then gently, but steadily, pulled away from the bar until both forepaws released. Peak force disturbance was automatically registered in grams-force (gf). Each mouse was tested five times in quick succession; the strongest measurement was taken as the score (reviewed by Crabbe et al. 2003). Grip strength was normalized by body weight (g) for statistical comparison.

### Open-field and light/dark box testing

Open field and light/dark box tests are based upon the rodent’s innate conflict between an aversion to exposed spaces and tendency to explore novel environments (Wallace et al. 2008; Cryan and Holmes 2005). The open field test measures changes in overall locomotion and may indicate states such as depression/anxiety that reduce willingness to explore. The test apparatus is a box (30 × 30 × 15 cm) divided into nine squares (Med Associates Inc., St. Albans, VT), enclosed in a larger, sound-attenuating box equipped with overhead lighting and ventilation. In the open-field test, a mouse naïve to the test apparatus was placed at its center and habituated for 1 min. Activity over 10 min was then monitored by 16 infrared beam sensors along the *X*–*Y* plane (Ramezani et al. 2011; Burger et al. 2005). In the light–dark box adaptation (Crawley and Goodwin 1980; Malmberg-Aiello et al. 2002), the test apparatus described above is separated into two equal compartments by a partition with an opening in the middle; a black plastic roof covers one compartment. Anxious animals tend to avoid exposure by spending more time in the dark (Bourin and Hascoet 2003). Each mouse was placed in the center of the lighted area (facing the darkened area) and allowed to explore the novel environment for 10 min. Activity was monitored as in the open field test. The number of squares crossed in both light and dark regions was recorded for 10 min. For both tests, data from 6-month-old mice that received 12 weeks of DOX treatment were normalized by scores from control age-matched groups fed normal chow.

### Stereology

Mice were deeply anesthetized with isoflurane (Baxter, Deerfield, IL, USA) prior to perfusion with 4 % paraformaldehyde (pH 7.4, Sigma-Aldrich Co., St. Louis, MO) in phosphate-buffered saline (PBS). After perfusion, brains were immediately removed and post-fixed in fresh fixative overnight, hemisected, rinsed several times, and left overnight in 15 ml of PBS. The left brain halves were coronally sectioned at 50 μm and stored individually in cryoprotectant [30 % sucrose (w/v), 1 % polyvinylpyrrolidone (v/v), 30 % ethylene glycol (v/v) in 0.05 M phosphate buffer, pH 7.2, all from Sigma] at −20 °C until use. Free-floating sections containing the striatum were stained with Hoescht 33342 (1 µg/1 ml, 8 min, Molecular Probes Inc., Eugene, OR) for all unbiased stereological estimation. Sections were thoroughly rinsed and then mounted on gelatin-coated SuperFrost Plus slides (VWR Scientific, West Chester, PA) in ProLong Gold anti-fade reagent (Life Technologies, Grand Island, NY, USA), and then air-dried for ≥8 h in the dark. The total number of (Hoescht^+^) cells in the striatum was estimated using the optical fractionator method (West et al. 1991); the Cavalieri principle was applied to measure striatal volume with assistance from a computerized stereology system (Stereologer, Stereology Resource Center, Chester, MD) (Mouton 2002). Every fifth section was selected from the total sections through the striatum and analyzed to estimate total cell number and striatal volume. Each reference space was outlined at low power (5×), and cells were counted using 100× magnification under oil-immersion. A guard volume of 2.0 μm was used during cell counting to avoid sectioning artifacts, including lost caps and uneven section surfaces. The slides were viewed on a Zeiss AxioObserver system with integrated Sony 3CCD Exwave HAD camera system (Carl Zeiss, Inc., Thornwood, NY).

### Immunohistochemistry and quantification

Perfusion and preparation of tissue sections were performed as described for stereology. 10 μm frozen or 50 μm free-floating sections were permeabilized with 0.2 % Triton X-100 in phosphate-buffered saline containing 1 % bovine serum albumin (Sigma-Aldrich) for 30 min. To assess proportional numbers of specific cells in the striata, single- or double-label immunostaining was performed. Primary antibodies specific for neuron-specific enolase (NSE; 1:100, Abcam, Cambridge, MA), neuron nuclei (NeuN; 1:200, Millipore, Temecula, CA), oligodendrocyte transcription factor 2 (Olig2; 1:100, Immuno-Biological Laboratories, Minneapolis, MN), aldehyde dehydrogenase family 1 member L1 (ALDH1L1; 1:500, Abcam), ionized calcium binding adaptor molecule 1 (Iba-1; 1:200, Wako, Osaka, Japan), and 3-nitrotyrosine (3-NT, 1:100, Santa Cruz Biotechnology, Inc., Santa Cruz, CA) were applied to sections. For double immunostaining, individual primary antibodies were sequentially applied and incubated overnight at 4 °C in a humidified chamber, followed by host-matched fluorescent-conjugated secondary antibodies (1 h, room temperature; Life Technologies). Immunostained sections were then incubated with Hoechst 33342 dye (1 μg/ml, 8 min; Life Technologies) to identify nuclei, rinsed thoroughly, mounted in ProLong Gold anti-fade reagent (Life Technologies), and then air-dried for ≥8 h in the dark. To verify the proportion of neurons, oligodendrocytes, astrocytes and microglia in the striata, 300–350 Hoechst^+^ cells were selected randomly per striatum and assessed for NSE, Olig2, ALDH1L1, and Iba-1 expression, respectively (*n* = 5–7). To determine the relative activation state of microglia, an independent group of 200 Iba-1^+^ cells were randomly selected per striatum and assessed for 3-NT expression. Striata were examined under oil immersion at 100× using a Zeiss AxioObserver system with integrated MRm camera system.

### Striatal neuron death

Neuron death was assessed by labeling for terminal deoxynucleotidyl transferase-mediated UTP nick end-labeling (TUNEL) followed by NeuN and Hoechst staining. A monoclonal antibody to NeuN and Hoechst 33342 (Life Technologies) were applied to 10 μm frozen coronal sections that included the striatum from all DOX treated groups as described above. TUNEL detection of in situ DNA fragmentation was performed in accordance with procedures in the In Situ Cell Death Detection Kit, TMR red (Roche Applied Science, Indianapolis, IN) in the same slides. To quantify active neuron death, the proportion of cells that were both TUNEL^+^ and NeuN^+^ was determined by counting at least 200 NeuN^+^ cells in adjacent fields in two different sections per animal (*n* = 6), at 63× magnification. To determine neuron death, 200 NeuN^+^ cells were randomly selected for assessment of TUNEL (*n* = 6).

### Immunoblotting

In vivo astrocytic markers and pre-/postsynaptic vesicle-associated proteins were examined by immunoblotting in striatal samples from all DOX treated groups (*n* = 6). Striata were freshly harvested and homogenized (T-PER Reagent, Thermo Scientific, Pittsburgh, PA), including a protease inhibitor cocktail (Roche Applied Science). Homogenized tissue lysates were centrifuged and then stored at −80 °C until use. Protein concentration of each sample was measured using the BCA protein assay (Pierce, Rockford, IL). 20 μg of lysates were loaded per well onto 4–20 % Tris–HCl Ready Gels (Bio-Rad Laboratories, Hercules, CA), and Precision Plus Protein Dual Color Standards (Bio-Rad; MW range 10–250 kDa) were used to visualize protein transfer and determine molecular weight. Proteins were transferred to PVDF membranes (Bio-Rad). Antibodies to astrocyte (ALDH1L1, 1:1000, Abcam; GFAP, 1:1000, Millipore) and pre-/postsynaptic [synaptotagmin 2 (Syt2), 1:100, Zebrafish International Resource Center; synapsin (Syn), 1:1,000, Synaptic Systems; gephyrin (Geph), 1:1,000, Synaptic Systems,; post synaptic density protein 95 (PSD-95), 1:3,000, Affinity BioReagents] were used to probe the blots. Anti-GAPDH (1:2,500, Abcam) was used to normalize protein loading. Host-matched IRDye^®^ Infrared Dyes-conjugated secondary antibodies (LI-COR Biotechnology, Lincoln, Nebraska) were applied to visualize each protein band. Protein bands were detected on an Odyssey^®^ Infrared Imaging System and intensity was analyzed by Odyssey 2.0 software (LI-COR).

### Morphology of striatal cells

Medium spiny neuron morphology was also assessed in tissues impregnated using a Golgi–Kopsch procedure (Hauser et al. 2009; Fitting et al. 2010b). Briefly, brain tissues were separately prepared from all groups after 10–16 weeks DOX administration (*n* = 6). Dendrites were assessed in tissues subjected to a modified Golgi–Kopsch procedure that impregnates cell processes of random neurons and glia in their entirety (Hauser et al. 1989, 2009). After 4 % paraformaldehyde perfusion as described above, whole forebrains were isolated and immersed in 2 % potassium dichromate and 5 % glutaraldehyde (v/v) in the dark at room temperature, and silver-impregnated as previously published (Fitting et al. 2010b, 2013). The ratio of dichromate solution to tissue volume was ≥50:1. After 6 days, tissues were gently washed in ultrapure water (3 × 1 min), tissues were gently blotted to remove excessive dichromate solution, and placed in aqueous 0.75 % silver nitrate for 5 days in the dark (50:1 fluid to tissue volume ratio). Intact forebrains were serial-sectioned on a vibrating microtome (Leica VT1200S, Leica Biosystems, Nussloch, Germany) at 110 μm in the coronal plane, dehydrated through graded ethanols, cleared in xylene, and mounted in Permount (Fisher Scientific, Waltham, MA). Wooden tools were used in all tissue handling. Several criteria were used to select dendrites for quantification: (1) the cell must be fully impregnated throughout its entirety (partially/incompletely impregnated cells were not assessed); (2) the dendrite must be parallel to the plane of the section, as tilted/angled dendrites can foreshorten the dendrite and influence density; (3) the dendrite must be distinct from dendrites on other neurons. Dendrites were considered pathologic when they were either stunted or extremely thin, or had spines that were unevenly distributed or stunted (Fig. 7A, E, F) (Fitting et al. 2010b; Ferrante et al. 1991; McNeill et al. 1988). The density of Golgi-impregnated spines was assessed on third order dendrites (3 dendrites per neuron; 6 neurons per mouse; *n* = 6 mice). In some neurons in male mice (but not females), all of the dendrites had gross decreases in spine density (≤5 spines per 10 mM) that were obvious even without counting (Fig. 7F). We purposely left these grossly abnormal neurons out of the measurements of average spine density so as not to unduly bias the results. Instead, those neurons were included in the counts of cells with pathologic dendrites (Fig. 7A).

### Electron microscopy

Male transgenic mice exposed to Tat for 2.5 months (*n* = 3) were perfused with 2 % paraformaldehyde/2 % glutaraldehyde in phosphate buffer (pH 7.4, Sigma-Aldrich Co.), and then post-fixed in 1 % osmium tetroxide (OsO_4_) for 1 h. For dehydration, the fixed tissues were processed through graded ethanols and infiltrated overnight in EMbed 812 (EMS, Hatfield, PA). Tissue was embedded in EMbed 812 and polymerized at 60 °C for 1–2 days. Thin sections of 600–700 Å thickness were cut on a Leica EM UC6i ultramicrotome (Leica Microsystems), collected onto formvar-coated grids, and stained with 5 % uranyl acetate and Reynolds’s lead citrate (Reynolds 1963). Sections were observed with a JEOL JEM-1230 TEM (JEOL USA, Inc.) at 2,000–12,000× magnification and images were obtained using a Gatan Ultrascan 4000 digital camera (Gatan Inc., Pleasanton, CA) with DigitalMicrograph™ software (Gatan Inc.).

### Statistical analysis

Analyses were done by one way, or main effect analysis of variance (ANOVA) followed by Duncan’s post hoc testing, and *t* test using Statistica 6.0 software (Statistica, Tulsa, OK). Student's *t*-test analyses were performed using GraphPad Prism 5 (GraphPad Software, Inc., La Jolla, CA).

## Results

### Accelerating rotarod assay

**Fig. 1:**
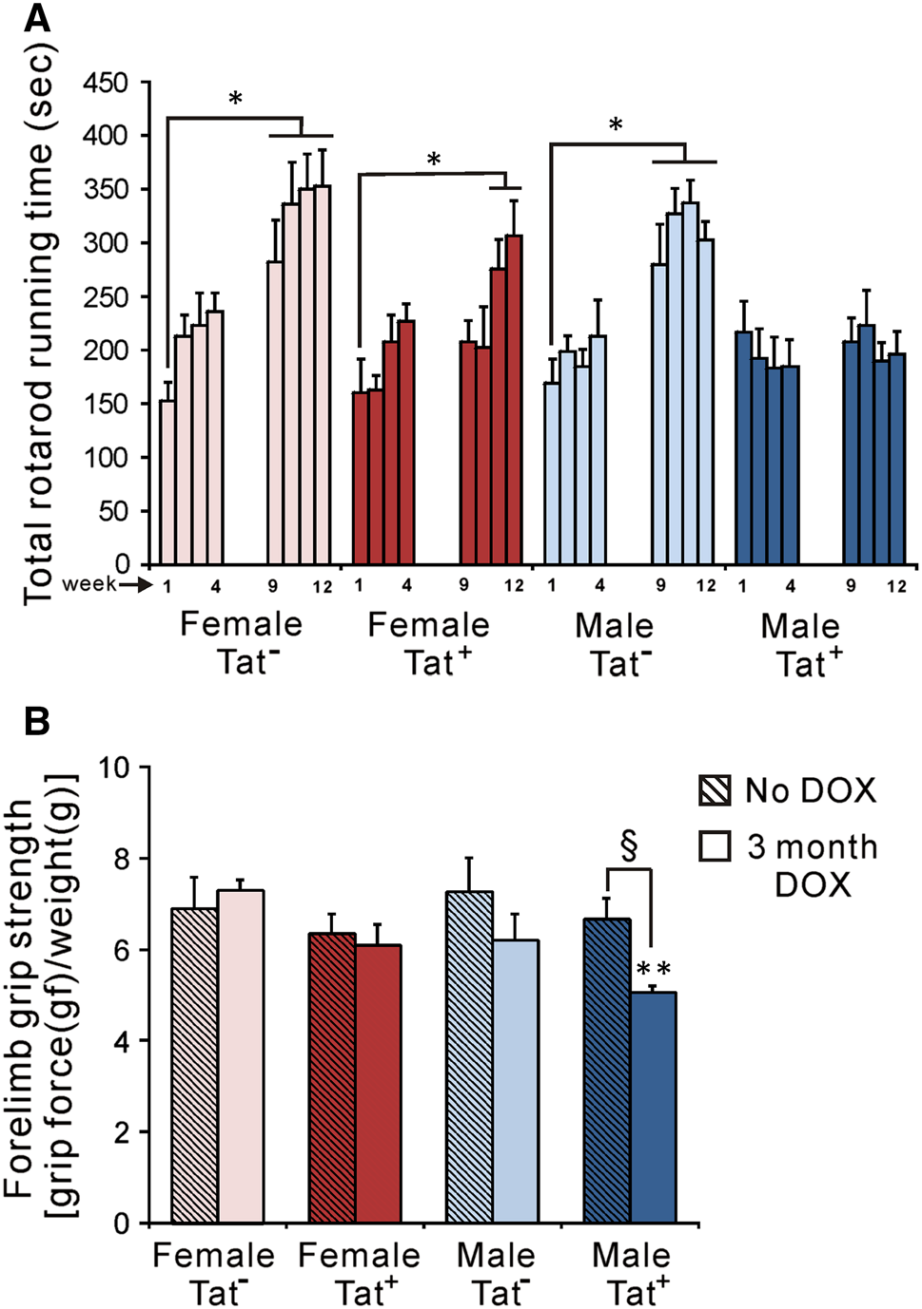
Chronic Tat expression in transgenic mice revealed a sex difference in Tat effects on motor acquisition and grip strength. Groups of Tat^−^ and Tat^+^ mice of both sexes were fed chow containing DOX to induce Tat expression continuously for 3 month in vivo. **A** Accelerating rotarod. All groups showed similar performance during weeks 1–4 of DOX exposure. By week 9 and thereafter, Tat^−^ mice of both sexes remained on the rotarod for a significantly longer time than at week 1. Female Tat^+^ mice showed a similar improvement by week 11. In contrast to all other groups, the performance of male Tat^+^ mice never differed from week 1. Thus, while Tat exposure affected rotarod performance in both sexes, there appeared to be a Tat-sex interaction on either task acquisition or motor ability that was specific to males (*p < 0.05 versus same mice at week 1, one-way ANOVA, Duncan post hoc test, *n* = 6–8). **B** Grip strength. Forelimb grip strength was tested in Tat^−^ and Tat^+^ mice of both sexes with/without DOX treatment. After 12 weeks, there was no difference due to sex or genotype among the groups fed normal chow. However, chronic exposure to HIV-1 Tat caused significantly decreased forelimb grip strength in the male Tat^+^ group compared with all others. Furthermore, male Tat^+^ mice were the only group affected by chronic Tat induction (^§^ *p* < 0.05; **p < 0.01, male Tat^+^ versus all other groups with DOX, one-way ANOVA, Duncan post hoc test, *n* = 6–8)

Mice exposed to DOX for 12 weeks performed an accelerating rotarod test once/week during weeks 1–4 and 9–12. All groups except for male Tat^+^ mice increased the duration of time spent on the rotarod over the 12 week period (Fig. 1A). Tat^+^ males remained on the bar for an average of <200 s at all times. Female and male Tat^−^ mice showed longer running times versus the initial trial at all times after week 4, reaching a maximum mean of 350 s. Female Tat^+^ mice reached a similar maximum running time but required more time to improve from their initial performance. There was also a significant difference between groups for the highest rotation speed reached before falling, which was 14 rpm for male Tat^+^ mice, and 21–24 rpm for all other groups (*p* < 0.05, one-way ANOVA, Duncan’s post hoc test, *n* = 6–8, data not shown).

### Forelimb grip strength

Forelimb grip strength was assessed in Tat^+^ and Tat^−^ mice fed DOX-containing chow for 12 weeks and in additional groups matched for age, sex, and genotype fed normal chow. Groups receiving normal chow showed no difference in forelimb grip strength after normalizing for body weight. DOX did not influence grip strength in females regardless of transgene expression (Fig. 1B), and Tat^−^ males receiving DOX had values equivalent to females. Forelimb strength was significantly reduced in male Tat^+^ mice receiving DOX (Fig. 1B), who showed the weakest grip strength among all groups receiving DOX .

### Open-field and light/dark box test

**Fig. 2:**
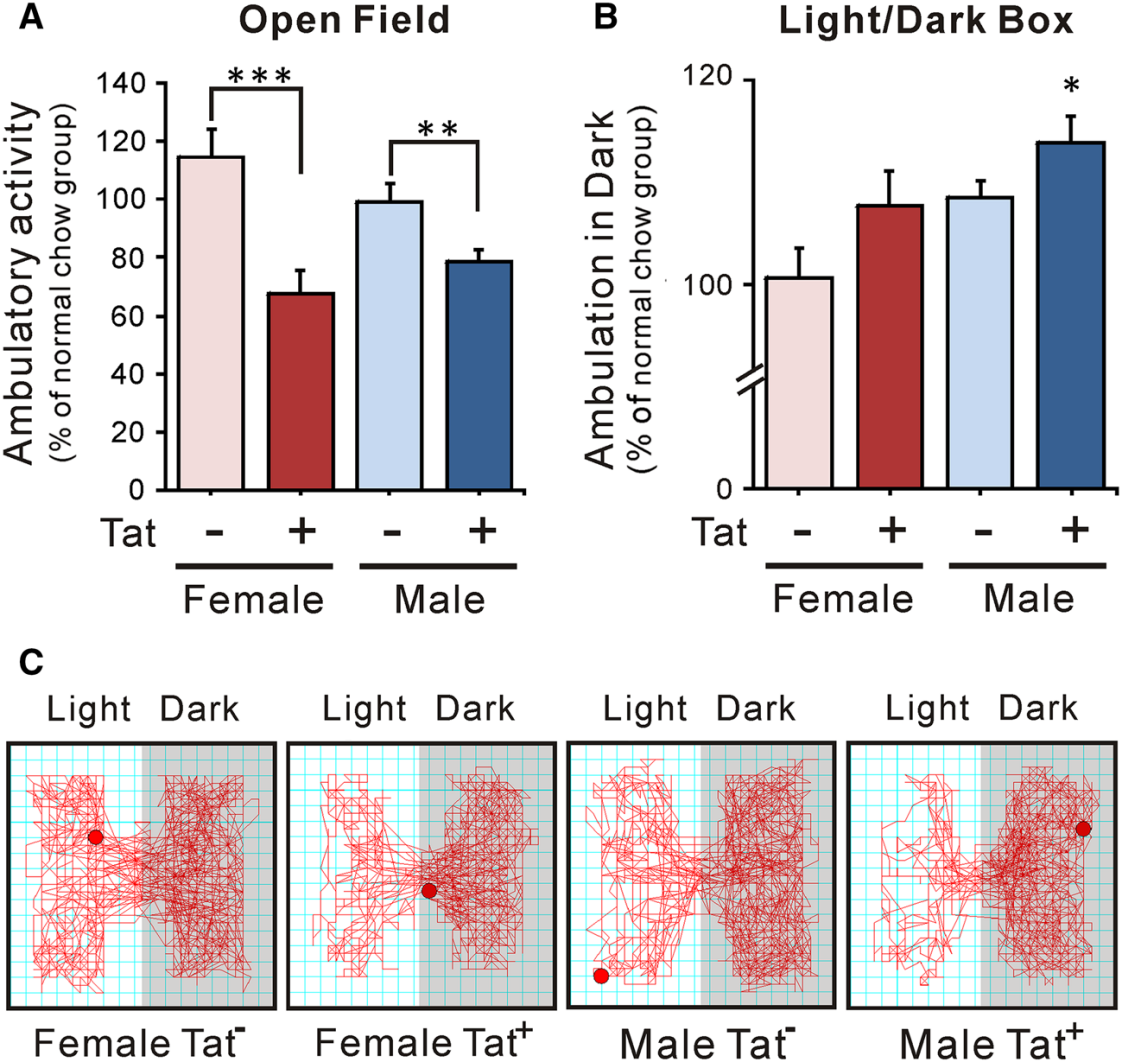
Long-term Tat exposure decreased ambulatory activity and induced anxiogenic-like behavior. Open-field and light/dark box tests were applied to Tat^−^ and Tat^+^ mice of both sexes at 6 months of age, after 12 weeks of normal or DOX-supplemented chow. Data for each DOX-treated group are presented as the percent of ambulatory activity normalized to the matched group receiving normal chow. **A** Open field test. Long-term Tat induction reduced ambulatory activity in both sexes (**p < 0.01; ***p < 0.001, one-way ANOVA, Duncan’s post hoc test, *n* = 6–8). There was no significant difference between female and male Tat^−^ or Tat^+^ mice. **B** Light/dark box test. Long-term Tat exposure selectively induced anxiogenic-like effects in the male Tat^+^ group compared to all other groups (*p < 0.05, one-way ANOVA, Duncan’s post hoc test, *n* = 6–8). **C** Representative diagrams of light/dark box test results. Tat^+^ males showed significantly increased ambulation in the dark side of the test compartment compared to all other groups

Open-field exploration of a novel environment was evaluated in Tat^+^ and Tat^−^ mice fed DOX-containing chow for 12 weeks and compared with age, sex, and genotype-matched groups fed normal chow. Total distance traveled in 10 min was significantly reduced in both male and female Tat^+^ mice after chronic Tat induction (Fig. 2A; Supplemental Table 1A), with no additional effect of biological sex.

A standard light/dark box test was performed to test whether HIV-1 Tat exposure induced anxiety-related behavior in a sex-dependent manner. Ambulation in the dark was similar for female Tat^−^ and Tat^+^ mice, and male Tat^−^ mice. However, male Tat^+^ mice had significantly higher ambulation in the dark compared with all other groups, suggesting an increase in anxiety-like behavior due to chronic HIV-1 Tat expression (Fig. 2B, C; Supplemental Table 1B).

### Stereological analysis of striatum cell number and volume

**Fig. 3:**
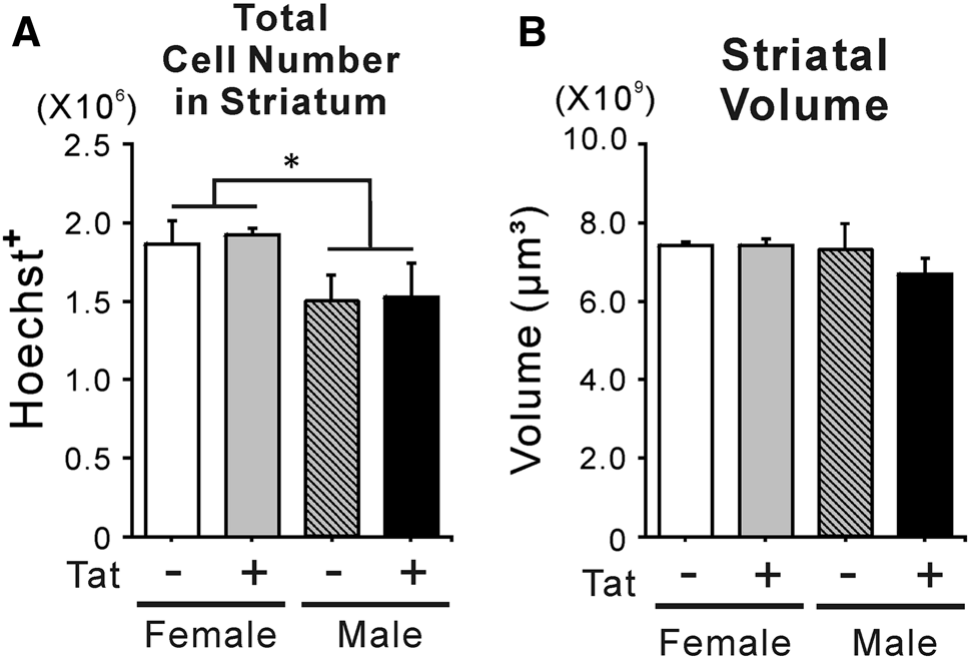
Effects of long-term Tat induction on total number of striatal cells and striatal volume. Brain sections including striata from Tat^−^ and Tat^+^ mice of both sexes at 6 month age, after 12 weeks of DOX-supplemented chow were utilized for stereological estimation of total striatal cell number and striatal volume. **A** Unbiased stereological estimates showed that total striatal cells (Hoechst^+^) were more numerous in female as compared to male mice (*p < 0.05, one-way ANOVA, main effect, *n* = 3). However, there was no effect of chronic Tat on total striatal cell number in either sex. **B** Striatal volumes were similar in males and females; chronic Tat induction by DOX administration did not alter the estimated striatal volume in either sex (*n* = 3)

The striatal sections examined were centered on the mid-caudate/putamen area, at approximately Section 25 in a mouse stereotaxic atlas (Franklin and Paxinos 1997). Unbiased stereological estimation of Hoechst-stained cells showed more total cells in the striatum of female than male mice irrespective of Tat expression (Fig. 3A), although there was no significant difference in the estimated striatal volume of any group (Fig. 3B).

### Effects of chronic Tat induction on total and TUNEL^+^ neuron populations in striata

**Fig. 4:**
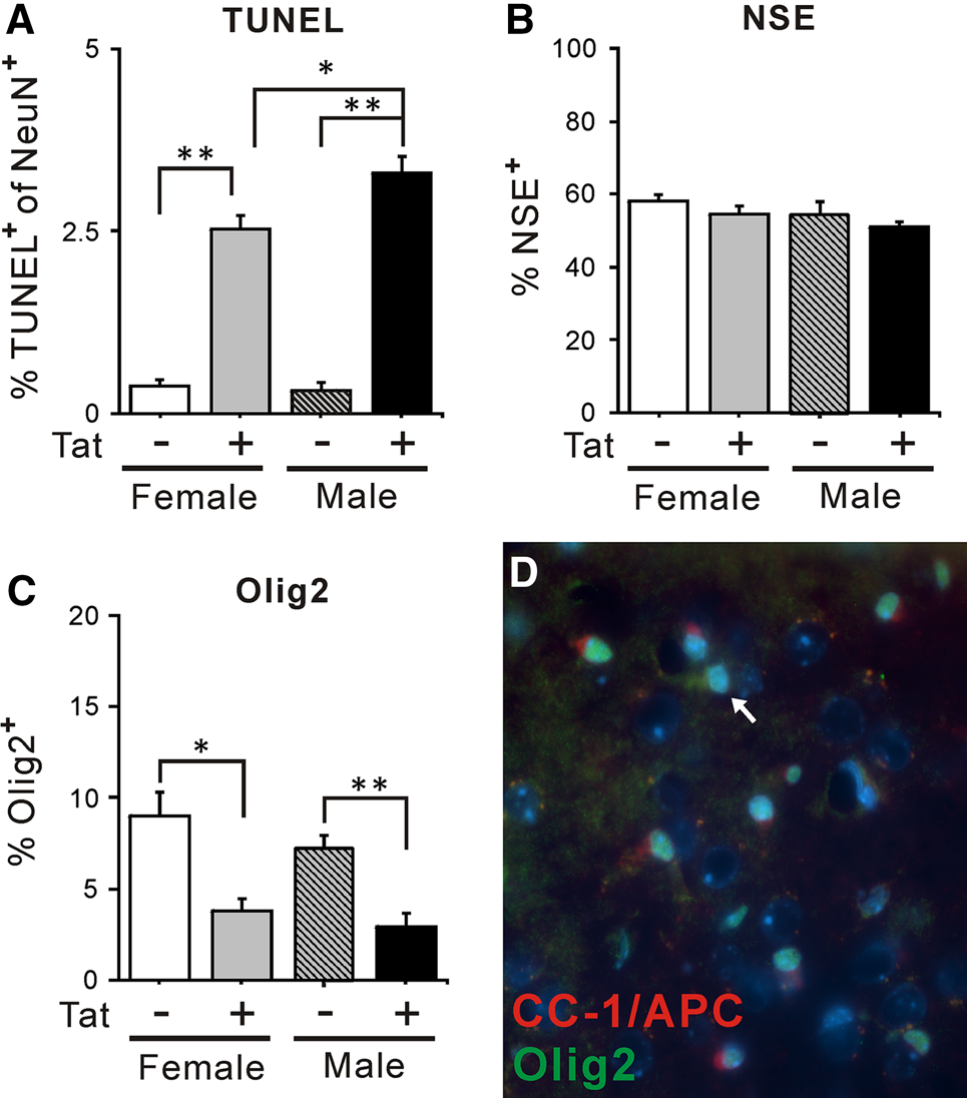
Effects of chronic Tat induction on neurons and NPCs in striata. Brain sections including the striatal region from DOX-treated Tat^−^ and Tat^+^ mice of both sexes were examined. **A** Chronic Tat expression increased the proportion of NeuN^+^ cells that were TUNEL^+^ in the striatum of both sexes (*p < 0.05, **p < 0.01, one-way ANOVA, Duncan post hoc test, *n* = 6–7). Additionally, male Tat^+^ striata had a greater percentage of TUNEL^+^/NeuN^+^ cells than were observed in female Tat^+^ striata. **B** The percent of NSE-immunostained cells was unaffected by Tat. Since there were no differences in striatal volume between the sexes, this suggests no difference in total striatal neuron numbers at this time (*n* = 5). **C** Chronic Tat induction also decreased the percent of cells expressing Olig2 in the striatum. The Olig2^+^ population was decreased similarly in Tat^+^ mice of both sexes (*p < 0.05, **p < 0.01, one-way ANOVA, Duncan post hoc test, *n* = 6). **D** The striatal cells were triple-labeled for Olig2^+^ (*green*), CC-1/APC^+^ (*red*), and the nuclear stain Hoechst 33342 (*blue*). Fluorescent imaging indicates that >90 % of Olig2^+^ cells are also CC-1/APC^+^.* White arrow* indicates an Olig2^+^ cell that does not express CC-1/APC. All other Olig2^+^ cells in this figure are double-labeled

Two neuron markers were used to assess the effect of chronic Tat induction on the striatal neuron population. Small increases in TUNEL reactivity were measured in both female and male Tat^+^ mice in NeuN^+^ and NSE^+^ (not shown) striatal neurons (Fig. 4A). Although the differences were significant, these TUNEL^+^/NeuN^+^ cells represented ≤3 % of all NeuN^+^ cells. The proportion of total cells (Hoechst^+^) that were NSE^+^ neurons was unaffected in either sex (Fig. 4B). NSE was used to assess total neurons, since immunostaining in these relatively thick sections was more consistent than with NeuN antibodies.

### Effects of chronic Tat induction on oligodendrocyte populations in striata

We examined the population of cells expressing the transcription factor Olig2 after 12 weeks of DOX treatment. Olig2 is a transcription factor expressed in oligodendrocytes at all developmental stages (Woodruff et al. 2001; Ligon et al. 2006). HIV-1 Tat induction significantly decreased populations of Olig2^+^ oligodendroglia in Tat^+^ females and Tat^+^ males with no significant differences due to sex (Fig. 4C). Double-labeling for Olig2 and the mature oligodendrocyte marker CC1/APC shows that >90 % of Olig2^+^ cells are also CC-1/APC^+^ (Fig. 4D).

### Effects of chronic Tat induction on astrogliosis

**Fig. 5:**
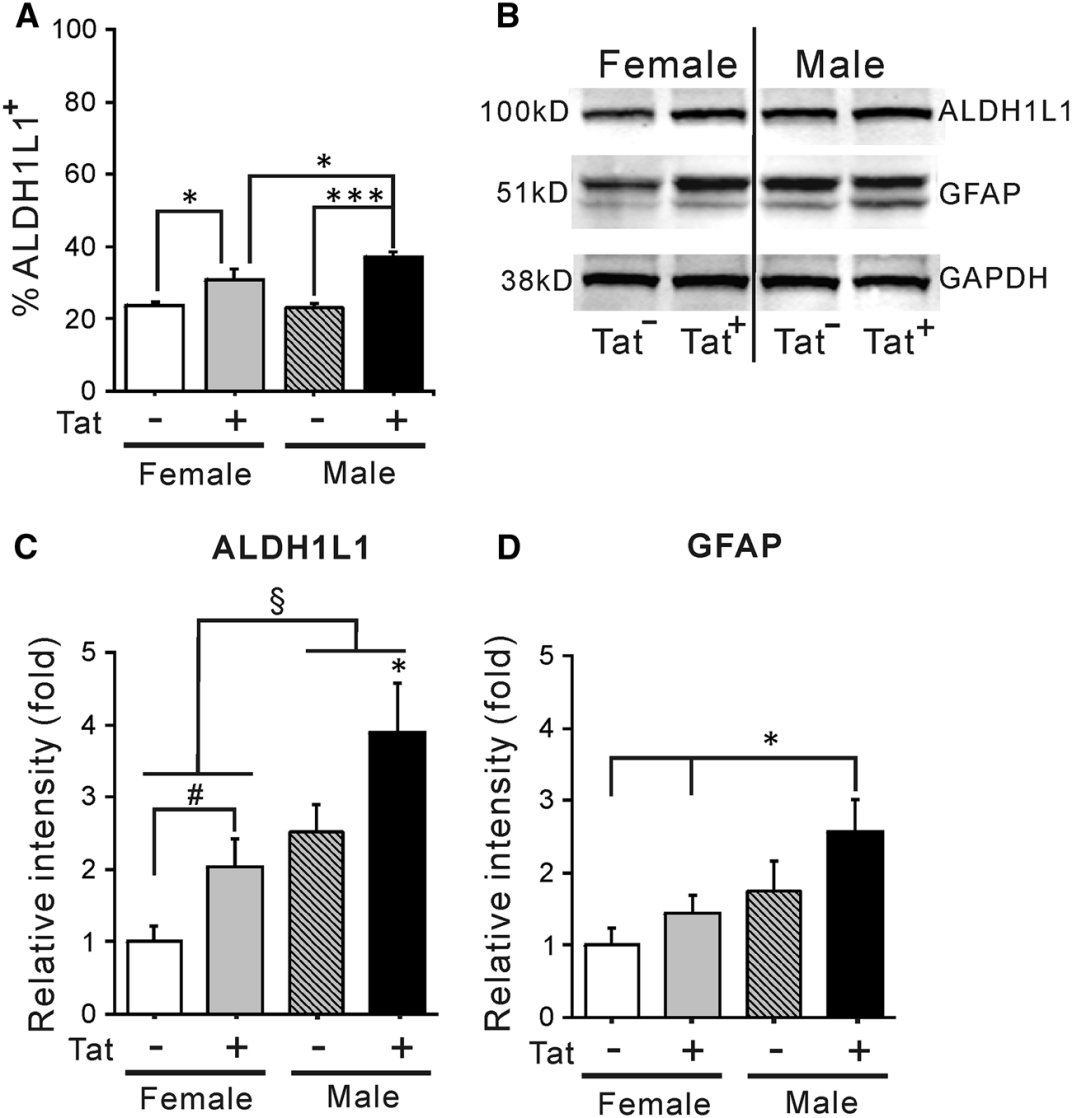
Effects of chronic Tat induction on striatal astroglia. **A** ALDH1L1 immunohistochemistry was performed on brain sections from all groups after 12 weeks of DOX administration. Chronic Tat exposure significantly increased the population of ALDH1L1^+^ cells in the striata of Tat^+^ mice of both sexes. The effect of Tat was significantly greater in Tat^+^ males than in Tat^+^ females (*p < 0.05; ***p < 0.001, one-way ANOVA, Duncan’s post hoc test, *n* = 6). **B**–**D** Immunoblots were also performed with ALDH1L1 and GFAP antibody after chronic Tat induction. Fresh striata were harvested and dissected from DOX-treated Tat^−^ and Tat^+^ mice of both sexes. **B** Shows representative blots for ALDH1L1, GFAP, and GAPDH. **C** The intensity of ALDH1L1 bands was significantly increased in tissue lysates from Tat^+^ mice of both sexes. Blots from lysates of male Tat^+^ mice were significantly more intense than those of any other group (*p < 0.05 versus all other groups, one-way ANOVA, Duncan’s post hoc test after normalization to GAPDH). The difference between Tat^+^ and Tat^−^ females was only detectable using a less stringent test (^#^<0.05, Student’s *t* test, one-tailed). Overall, male Tat^+^ lysates showed greater ALDH1L1 intensity than female Tat^+^ lysates after normalization (^§^ *p* < 0.05, one-way ANOVA, main effect, *n* = 6). **D** The effects of Tat were less robust when GFAP was examined, but there was a sex-related interaction. Tat^−^ mice of both sexes had equivalent, normalized GFAP expression. Chronic Tat induction by DOX did not increase GFAP levels in either male or female mice versus their Tat^−^ controls. However, after 12 weeks of DOX administration, the intensity of GFAP immunoblots in male Tat^+^ mice was greater than both Tat^+^ and Tat^−^ female mice (*p < 0.05, one-way ANOVA, Duncan’s post hoc test)

To assess Tat-induced changes in the proportion of astrocytes in striatum, we used two reliable markers of astroglia, GFAP and aldehyde dehydrogenase 1 family, member L1 (ALDH1L1), which may be a more reliable marker in rodent brain (Yang et al. 2011; Cahoy et al. 2008). The proportion of total (Hoechst^+^) striatal cells expressing ALDH1L1 was increased for both Tat^+^ males and females after 12 weeks of DOX treatment. Additionally, there was a sex effect since male Tat^+^ mice had significantly more ALDH1L1^+^ cells compared with female Tat^+^ mice (Fig. 5A). After chronic Tat induction, the intensity of ALDH1L1 bands in immunoblots from tissue lysates of both female and male Tat^+^ mice was increased (Fig. 5B). Those from male Tat^+^ mice were significantly enhanced compared with all other groups (Fig. 5C), suggesting a sex-related interaction on gliosis. GFAP band intensity in Tat^+^ groups was not significantly increased versus the Tat^−^ group of the corresponding sex, although Tat^+^ male mice did have higher GFAP levels than female mice of either genotype. Overall, even considering the differences between GFAP and ALDH1L1 results, male mice show higher astrocyte activation than females (Fig. 5B-D).

### Effect of chronic Tat induction on reactive microglia

**Fig. 6:**
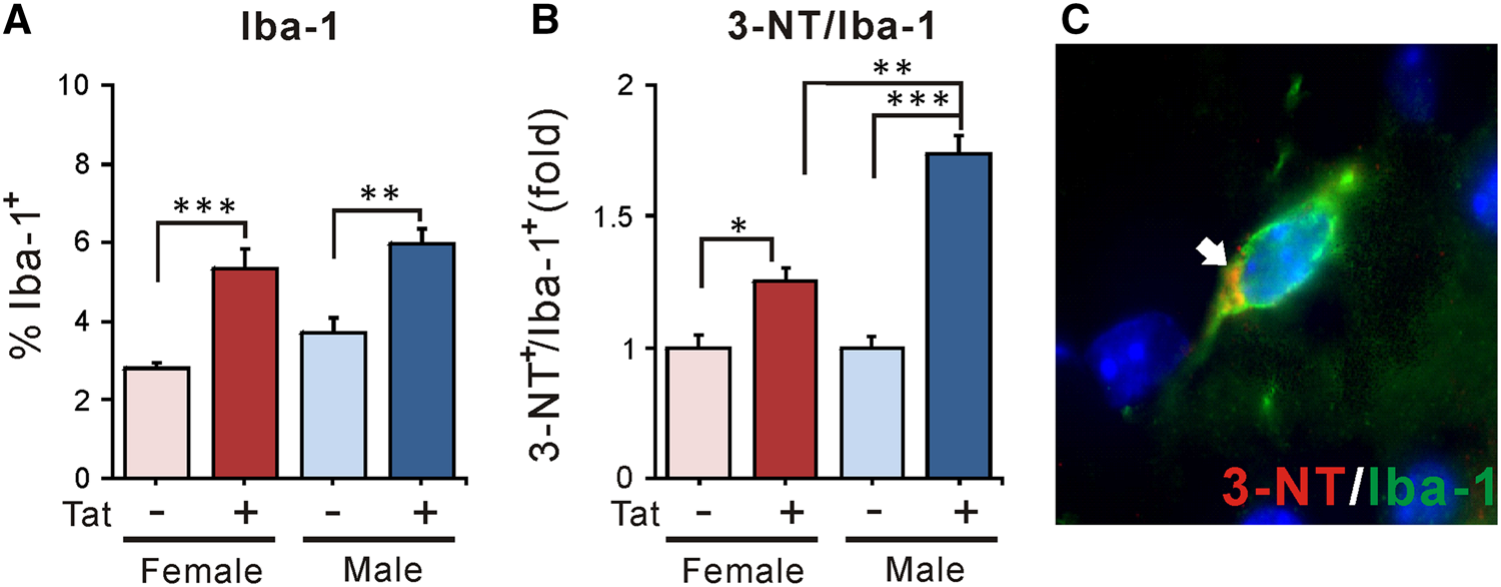
Effects of chronic Tat induction on microglia in striatum. **A** The proportion of striatal cells expressing Iba-1 was increased to the same extent in both chronically induced male and female Tat^+^ groups compared with their respective Tat^−^ controls (**p < 0.01; ***p < 0.001, Duncan’s post hoc test, *n* = 6–7). **B** Chronic HIV-1 Tat exposure also induced a significant increase in the production of 3-NT in Iba-1^+^ cells of both sexes. Although there was no sex difference in Tat effect on the Iba-1^+^ population overall, 3-NT expression within the Iba-1^+^ population was significantly higher in male Tat^+^ mice than in female Tat^+^ mice (*p < 0.05; **p < 0.01; ***p < 0.001, Duncan’s post hoc test, *n* = 6–7). Thus, HIV-1 Tat exposure appears to enhance microglial numbers equally in both sexes, but to disproportionately enhance the production of nitrosylated species in microglia within male mice. **C** Fluorescence image of a triple-labeled, Iba-1^+^ (*green*)/3-NT^+^ (*red*) microglial cell. *Blue color* indicates Hoechst 33342 staining within the nuclei of this cell, and nearby cells that are not Iba-1^+^. 3-NT immunostaining within the scant microglial cytoplasm is indicated by the *arrow*. Note the ramified appearance of the microglial cell, whose processes likely branch out of the field of this 10-μM section. Microglia with a ramified morphology are usually associated with lower levels of activation, and it is interesting that even cells with this morphology have detectable 3-NT

After 12 weeks DOX treatment, the percent of Iba-1^+^ cells among total striatal cells of female and male Tat^−^ mice was similar, and Tat induction caused comparable increases in the proportion of Iba-1^+^ cells (Fig. 6A). 3-NT, a product of tyrosine nitrosylation mediated by reactive nitrogen species such as peroxynitrite (ONOO^−^) and nitrogen dioxide (NO_2_), has been used as a marker of nitrosative cellular stress (Ryu and McLarnon 2006; Shavali et al. 2006; Shishehbor and Hazen 2004), particularly in activated microglia. Iba-1/3-NT co-localization was assessed as a measure of microglial activation. Tat induction significantly increased the proportion of microglia expressing 3-NT in both sexes (Fig. 6B, C). There was a significant interactive effect of sex since the proportion of 3-NT^+^ microglia was higher in male than in female mice (Fig. 6B).

### Effect of chronic Tat induction on spine density, morphology, and synaptic proteins

#### Morphology

**Fig. 7:**
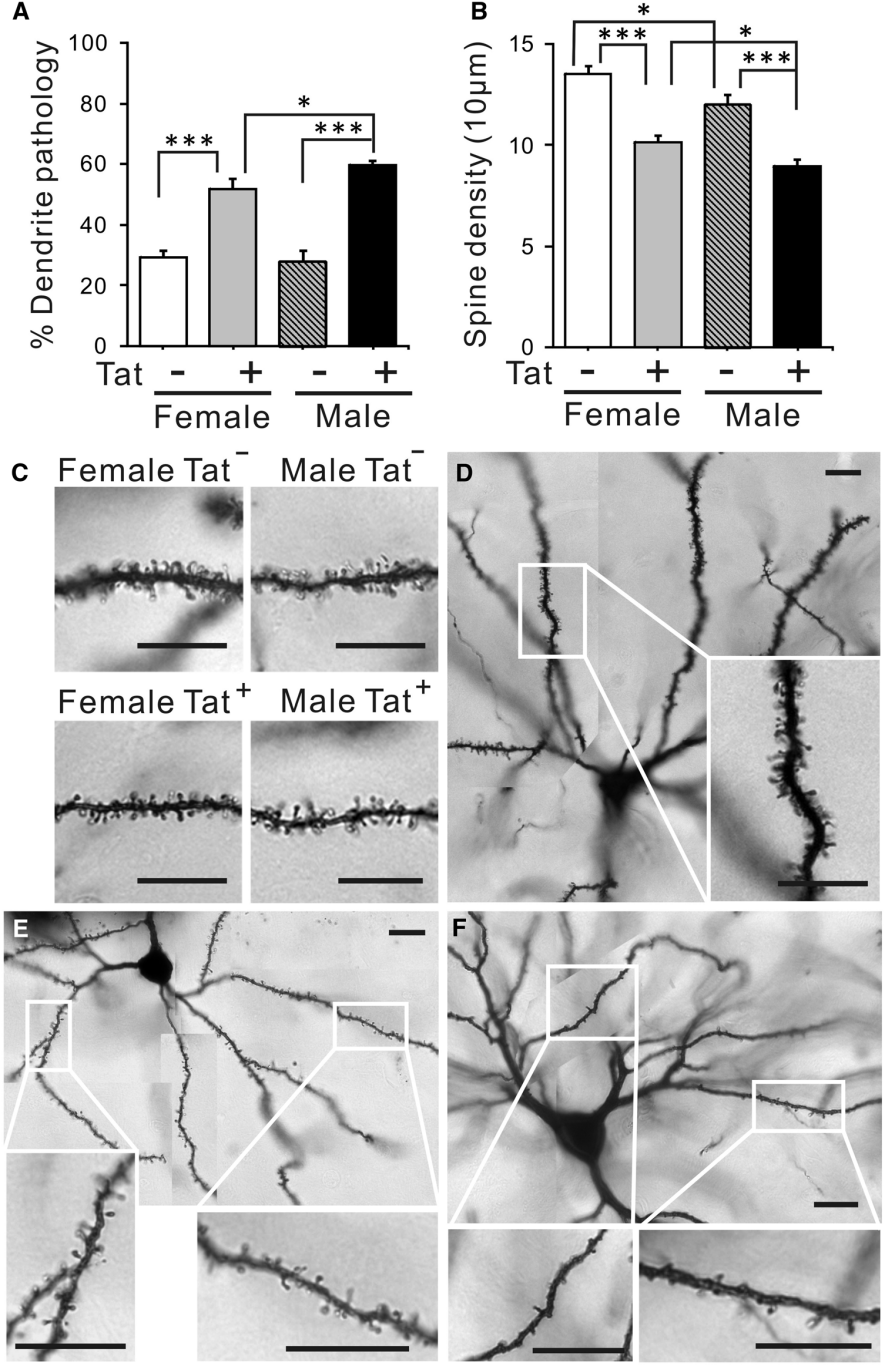
Effects of chronic Tat induction on spine density and dendrite morphology in striatal medium spiny neurons. **A** Each neuron was categorized as having dendrites with an entirely normal morphology, or having an abnormal morphology with one or more dendrites that displayed aberrant morphology, such as uneven spine distributions, low spine number (less than 5 in 10 μm), or stunted or extremely thin dendrites. The proportion of neurons that possessed one or more dendrites with abnormal morphology was counted and reported as a percentage of total neurons examined (20 neurons per mouse, each with ≥3 dendrites, 6 mice). Chronic Tat induction significantly increased the percentage of neurons with morphological abnormalities in both sexes. Male Tat^+^ mice were significantly more affected, suggesting a Tat-sex interaction (*p < 0.05, ***p < 0.001, one-way ANOVA, Duncan’s post hoc test, *n* = 6). **B**, **C** Dendritic spines were counted on third-order dendrites of medium spiny neurons. Density was recorded as the mean number of spines per 10 μm dendrite length, averaged for each animal (spines/10 μm) (*n* = 6 mice; 3 dendrites per neuron; 6 neurons per mouse). The baseline spine density in Tat^−^ mice showed a slight but significant sex-specific difference, with lower density in males. Tat induction significantly decreased total spine density in Tat^+^ mice of both sexes as compared with Tat^−^ mice (*p < 0.05, **p < 0.01, ***p < 0.001, one-way ANOVA, Duncan’s post hoc test, *n* = 6). *Scale bars* 10 μm. **D** Normal medium spiny neuron in striatum of Tat^−^ male. **E**–**F** Medium spiny neurons with abnormal morphologies in the striatum of Tat^+^ males. The cells in **E** have sparse spines that unevenly distributed; the cell in **F** has extremely few spines, most of which are very stunted. Cells like that shown in **F**, in which all dendrites were abnormal, were only found in Tat^+^ male mice and represented less than 5 % of all impregnated striatal neurons. This type of cell was never found in other groups, including Tat^+^ females. Insets in **D** through **F** show higher magnification of areas indicated in larger figures

A significant increase of medium spiny neurons with pathologic dendrites was observed in Tat^+^ mice of both sexes, with a slightly greater increase in males versus females (Fig. 7A). This category includes stunted or extremely thin dendrites or those having spines that were unevenly distributed or stunted (Fig. 7E, F). It also includes neurons in which all visible, third-order dendrites had severe spine loss (density ≤5/10 μM; Fig. 7E, F).

#### Spine density

For each neuron examined, the density of spines was measured on all visible third-order dendrites. Adult mice normally exhibit spine densities of approximately 15 per 10 μM in such dendrites (Cheng et al. 1997). In 2–5 % of neurons in Tat^+^ mice, all visible dendrites had a spine density of less than 5 per 10 μM (Fig. 7F); this level of pathology, which was obvious even without counting, was never seen in Tat^−^ mice. To obtain a more accurate assessment of spine density in the seemingly normal dendrites, neurons with these extremely spine deficiencies were not included in the calculation of average spine densities. Even exclusive of the severely aberrant neurons, average spine density was decreased in Tat^+^ mice. There were significantly fewer spines in males regardless of genotype (Fig. 7B, C).

#### Synaptic proteins

**Fig. 8:**
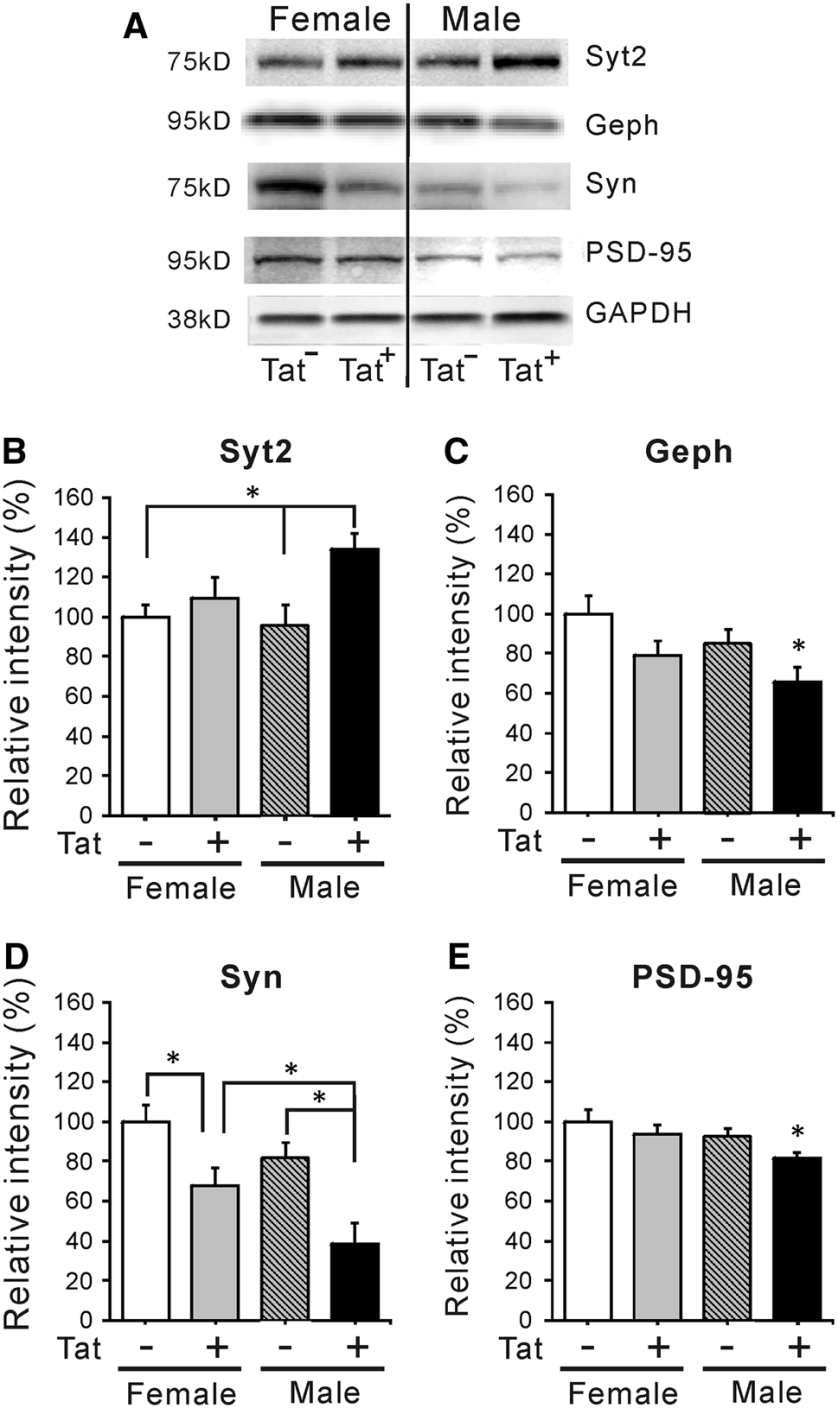
Effects of chronic Tat induction on expression of pre- and post-synaptic markers in striatum. **A** Striatal lysates were examined by immunoblotting to quantify levels of protein pairs associated with inhibitory (Syt2, gephyrin) and excitatory (Syn, PSD-95) pre- and post-synaptic membranes, respectively. The intensity of all bands was normalized to GAPDH. **B**, **C** Levels of inhibitory synaptic proteins were only affected in male Tat^+^ mice. Relative to most other groups, presynaptic Syt2 was increased (*p < 0.05, one-way ANOVA, Duncan’s post hoc test, *n* = 6) and postsynaptic gephyrin (Geph) was decreased (*p < 0.05 versus all other groups, one-way ANOVA, Duncan’s post hoc test, *n* = 6). **D**, **E** Both female and male Tat^+^ mice exhibited a decrease in the excitatory presynaptic protein synapsin 1 (Syn) compared with Tat^−^ mice; levels in male Tat^+^ mice were significantly less than those in female Tat^+^ mice. The excitatory postsynaptic PSD-95 protein was reduced only in male Tat^+^ mice, compared with all other groups. (*p < 0.05, **p < 0.01, one-way ANOVA, Duncan’s post hoc test, *n* = 6)

Changes in spine density are likely to affect synaptic organization and function; thus we performed immunoblot analysis to determine if Tat induction affected the stability of either pre- or post-synaptic inhibitory (Syt2, gephyrin) and excitatory (Syn, PSD-95) synaptic proteins. Tat induction altered levels of both inhibitory synaptic proteins, but only in brains of male mice, where Syt2 was increased and gephyrin was reduced (Fig. 8A–C). The excitatory presynaptic Syn protein was decreased in both sexes of Tat^+^ mice; as observed for other parameters including spine density, males were significantly more affected (Fig. 8A, D). Excitatory postsynaptic PSD-95 levels were specifically reduced in Tat^+^ males (Fig. 8A, E).

### Striatal ultrastructure

**Fig. 9:**
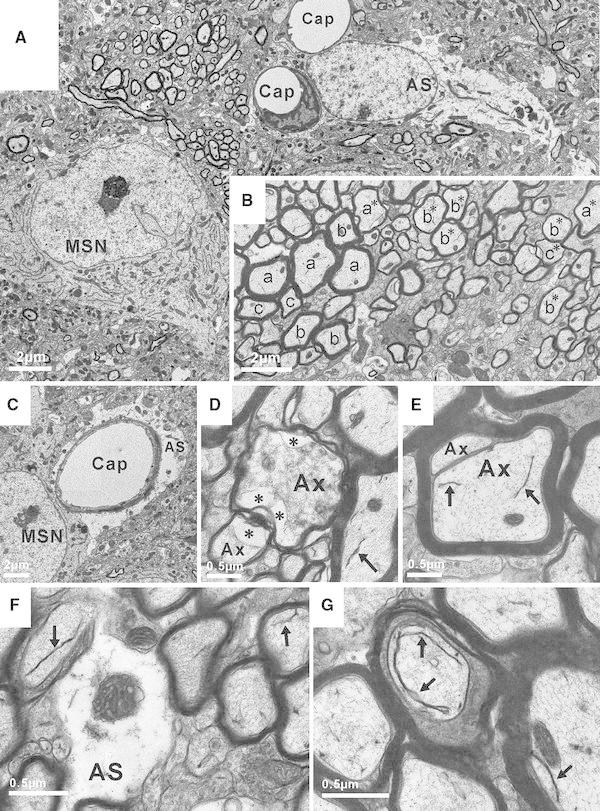
Ultrastructure of the striatum after 3 months of Tat induction in transgenic mice. Although much of the neuropil appears normal, both glia and neurons exhibit multiple features that are atypical. **A** A montage view shows the healthy-appearing nucleus and cytoplasm of a medium spiny neuron (MSN), two capillaries (Cap), one of which has an associated endothelial cell, and surrounding neuropil of normal density. Axons all appear to have myelin of an appropriate thickness. While the astrocyte (AS) has a seemingly normal nucleus with nucleoplasm of homogeneous density and a relatively inconspicuous nucleolus, the cytoplasm of the astrocyte is abnormally clear and watery and does not contain the intermediate filaments that usually distinguish this cell type. **B** Abnormal myelination in a group of axons. In the normal adult CNS, myelinated axons of the same caliber are invested with myelin sheaths that are very similar in thickness within a given brain region. This “g-ratio” (axon diameter/fiber diameter) is used to assess normal myelination. Note that while most axons towards the left side of the field are appropriately myelinated, many axons of equal caliber towards the right side have thin myelin. The labels “*a*–*c*” indicate groups of axons of decreasing caliber with myelin sheaths of relatively normal thickness; labels “a*–c*” indicate similar groups of axons that have much thinner myelin than their counterparts in the “*a*–*c*” groups. The myelin is of good compaction, indicating that if remyelination has occurred, the process has not been recent. **C** A neuron (MSN) with normal nucleoplasm and cytoplasm is found next to a capillary (Cap) that is invested with a large amount of watery astroglial cytoplasm (AS). It is normal to have astroglial endfeet enveloping a capillary, but this large volume of swollen cytoplasm is not normal. Capillaries with similarly distended astroglial endfeet are common in the tissue. **D** Two of the axons (Ax) in this group show clear signs of degeneration. The integrity of the axoplasm has been compromised, and there is also obvious shrinkage/contraction of axoplasmic area. In comparison with adjacent normal axons, very few neurofilaments can be distinguished within the axoplasm, which is of an unusual, flocculent consistency. *Asterisks* indicate regions where the axoplasm has retracted from the surrounding myelin sheath. Degenerating axons are occasionally seen within the normal CNS, but are more prominent in these tissues. *Arrow* indicates an abnormal membrane structure discussed in **F** and **G**. **E** A myelin sheath has improperly formed around two axons (Ax). At higher magnification (not shown), the axons clearly are contained within separate outer membranes. *Arrows* indicate abnormal membranous bodies that are frequently seen in these axons (see **F**, **G**). **F** This panel shows a higher magnification view of unusually watery astroglial cytoplasm (AS), as well as two examples of abnormal membrane structures that are frequently seen within axons from these Tat^+^ transgenic mice (*arrows*). These membrane bodies can reach quite remarkable lengths (see **G**) and in some cases appear to be multilamellar. They are quite different in structure from smooth endoplamic reticulum, and at present their origin is undetermined. They have not been observed in axons in the striatum or hippocampus of Tat^+^ transgenic mice after 7–10 days of DOX exposure (Fitting et al. 2010b, 2013), nor are they observed in Tat^−^ mice receiving DOX for the same 3 month period (not shown). **G** An axon contains a dense, ungranulated membranous structure of remarkable length (*double arrows*) within otherwise normal-appearing axoplasm. This membranous body appears to be multilamellar. The large amount of oligodendroglial cytoplasm surrounding the axon is not necessarily pathologic. A similar but smaller membranous structure is indicated by an *arrow* in an adjacent axon. *Bars* in each panel indicate the magnification

Qualitative electron microscopic evaluation revealed several structural anomalies in Tat^+^ male tissue (Fig. 9). Most prominent were seemingly normal capillaries invested by notably swollen astroglial cytoplasm. Expanded, watery astroglial processes were conspicuous throughout an otherwise normal appearing neuropil. The cytoarchitecture of neuron cell bodies appeared largely normal, with perhaps more frequent autophagic inclusions. Axonal damage was noted, as were areas of axons with abnormally thin myelin. Cellular ultrastructure in Tat^−^ mice appeared normal.

## Discussion

In the post-cART era, neuroAIDS has become a chronic disease largely characterized by an increased prevalence of milder cognitive and motor disorders (HAND) with less encephalitis (Antinori et al. 2007; McArthur et al. 2010; Sacktor et al. 2001; Gonzalez-Scarano and Martin-Garcia 2005; Woods et al. 2009; Ellis et al. 2007; Wojna et al. 2006; Dore et al. 2003; Lawrence and Major 2002; Grant et al. 1995; Heaton et al. 1995). Although the subset of female HIV patients has expanded, the question of whether sex influences the occurrence and characteristics of neurocognitive dysfunction in HIV^+^ patients is largely unexplored. Studies on HAND epidemiology in western countries, where the disease is more prevalent in men, focus largely on males. In more resource-limited settings, social variables between the sexes that affect health care access and quality make sex-related disease vulnerabilities difficult to determine. A recent study suggests that overall rates of neuropsychological impairment may not reflect the entire picture; HIV^+^ men and women may be differentially impaired in certain attention/memory tasks yet show a similar overall decline (Failde-Garrido et al. 2008).

Inducible Tat-transgenic mice are a reasonable model for testing whether sex influences HAND development since they exhibit many neuropathologic and behavioral deficits seen in HIV patients with HAND, including gliosis and microglial activation (Bruce-Keller et al. 2008), dendritic abnormalities and reduced dendritic spine density (Fitting et al. 2010b, 2013; Hauser et al. 2009), disrupted hippocampal circuitry, changes in synaptic proteins (Fitting et al. 2013), and learning/behavioral deficits (Fitting et al. 2013; Carey et al. 2012). The transgenic line used here, developed by Dr. Avi Nath, appears to exhibit less CNS pathology than a similar line (Zou et al. 2007; Kim et al. 2003) and was chosen because the pathology has a slower onset more representative of that in HIV patients. While most in vivo studies have used HIV-1 Tat exposure times of ≤2 weeks, we chose a 3-month exposure as potentially more reflective of persistent human disease. In a variety of well-characterized animal behavior tests, chronic Tat exposure negatively affected motor, cognitive, and anxiety measures, in general agreement with outcomes after more acute exposure (Fitting et al. 2013; Carey et al. 2012, 2013), and mirroring cortical and subcortical functional deficits in HIV patients that affect both cognitive and motor skills (Woods et al. 2009; McArthur et al. 2010; Heaton et al. 2010). Importantly, our results also provide initial evidence of an underlying difference in male versus female vulnerability. In all instances where severity differed between sexes, Tat^+^ male mice had poorer outcomes, while Tat^+^ females were similar to control/Tat^−^ mice.

CNS tissue was evaluated to determine if cell populations were altered in a similar sex-specific manner, perhaps accounting for sex-specific behavioral changes. Since multiple brain regions are undoubtedly involved in these quite varied behavior deficits, we specifically examined the dorsal striatum, which is clearly impacted by HIV, and as the main input region for the basal ganglia is also involved in coordinating multiple behaviors. While the striatum is well known to control and modulate movements and tasks involving motor memory, striatal integration is also a key component in executive functions and decision making, and in processing motivational and emotional information relating to reward and anxiety (Voytek and Knight 2010; Balleine et al. 2007; Helfinstein et al. 2012). Unbiased stereological analyses indicated that Tat exposure did not affect either striatal volume or total striatal cell number in either sex. When individual populations of cells were quantified, there were effects of both sex and Tat exposure (Figs. 4, 5). Tat expression increased the percentage of apoptotic (TUNEL^+^) neurons, with slightly greater effect on male brains. The increased neuron loss was not reflected in total NSE^+^ numbers. We interpret this to suggest that neurons may only have started to die relatively recently, which is in keeping with our finding that TUNEL^+^ striatal neurons were not increased after short-term Tat exposure in these mice (Bruce-Keller et al. 2008). Over time, if a low rate of neuron death continues in the absence of replacement, the neuron population will eventually be reduced by a significant level . Tat expression also reduced Olig2^+^ oligodendroglia independent of sex, perhaps relating to reports that HIV-1 Tat reduces young oligodendrocyte proliferation in vitro (Hahn et al. 2012).

In the absence of neuron loss, what might account for observed behavioral deficits? Since we only examined total neurons, changes in subpopulations of striatal neurons affecting behavior may have been overlooked. Another possibility relates to changes in glial populations (Figs. 5, 6). Chronic exposure to Tat expanded the CNS population of ALDH1L1^+^ astroglia, as previously observed for GFAP^+^ astroglia after acute Tat exposure (Bruce-Keller et al. 2008). Cell counts and immunoblots generally concurred that astroglial expansion/astrogliosis was greater in males. As in more acute studies (Gupta et al. 2010; Hahn et al. 2010; Fitting et al. 2010c; Suzuki et al. 2011; Bruce-Keller et al. 2008), the microglial population also increased after chronic Tat exposure. Both sexes showed equivalent Iba-1^+^ cell increases, but 3-NT, a nitrosative product indicative of microglial activation, was specifically elevated in males. Thus, both microglial activation and astrogliosis correlate with altered behavior in male Tat^+^ brains. While not proving a causal relationship, the sustained, low levels of inflammation described in HIV^+^ brains even after cART treatment do produce cumulative deficits (Heaton et al. 2010, 2011; Cohen et al. 2010; Gongvatana et al. 2013) and would predict this outcome. Additionally, or alternatively, the relative deficits in male behavior might derive from their more significant abnormalities in dendrite structure and levels of specific synaptic proteins (Figs. 7, 8). Chronic Tat exposure reduced the density of spines on striatal medium spiny neurons in both sexes, similar to other reports with Tat, gp120 or HIV (Sa et al. 2004; Fitting et al. 2010b; Viviani et al. 2006). The pattern of loss was remarkably similar to spine changes reported with cortical deafferentation (Cheng et al. 1997), suggesting interruption of normal cortical connections that account for over 80 % of striatal input. The fact that spine loss recovers after a deafferentation injury (Cheng et al. 1997) may be a hopeful sign for the recovery potential of HIV patients. Both spine loss and abnormal dendrite pathology were clearly higher in male mice, which also showed changes in proteins associated with both inhibitory (presynaptic Syt2, postsynaptic gephyrin) and excitatory (presynaptic synapsin, postsynaptic PSD95) synapses. In females, only the excitatory presynaptic protein synapsin was modestly reduced. While HIV exposure may reduce synaptodendritic efficacy in general (Masliah et al. 1997; Everall et al. 1999; Fitting et al. 2013), Tat interactions may specifically affect how efficiently inhibitory versus excitatory information is processed, at least in striatum (Figs. 7, 8) and hippocampus (Fitting et al. 2013). Sex-specific protein alterations may thus contribute to differences in behavioral outcomes. Notably, reversible changes in excitatory synaptic proteins have been reported in vitro (Shin and Thayer 2013; Shin et al. 2012). Changes in synaptic efficiency likely occur before neuron loss, since synaptic protein changes occurred in hippocampus after more acute (10 days) Tat exposure (Fitting et al. 2013).

An extensive literature, both epidemiological and experimental, suggests that males are more vulnerable to trauma and neurodegenerative events/diseases (de Lau and Breteler 2006; Van Den Eeden et al. 2003; Coronado et al. 2011; Mehal et al. 2013). The lower vulnerability of females may be related to the documented neuroprotective role of estrogen in trauma and neurodegenerative studies (Yune et al. 2008; Rau et al. 2003; Roof and Hall 2000; D’Astous et al. 2004; Hoffman et al. 2006; Tang et al. 1996), although recent work emphasizes that sex as an injury variable includes more than single hormone-single receptor interactions, and involves both genomic and non-genomic effects (Cheng and Hurn 2010; Herson et al. 2009). Although a preponderance of evidence suggests that some component of estrogen-mediated protection derives from down-regulation of dopamine transporter function in the striatum (Wallace et al. 2006; Disshon and Dluzen 1999; Murray et al. 2003), other mechanisms are likely involved in striatum and elsewhere, including expression of endogenous anti-oxidants such as paraoxonase 2 (Giordano et al. 2013) and glutathione (Kumar et al. 2011), as well as anti-oxidant enzyme systems (Kumar et al. 2011; Rao et al. 2011). Recent evidence also suggests that a component of estrogen-mediated neuroprotection may be due to promotion of adaptive responses within neurons, and that males and females can respond differently to locally produced steroids versus those produced or delivered systemically (Gillies and McArthur 2010). Estrogens or ER-β signaling has been shown to exert neuroprotective effects on several HIV models including gp120 injection (Corasaniti et al. 2005), and in vitro Tat or gp120 exposure (Russo et al. 2005; Adams et al. 2010, 2012; Wallace et al. 2006; Brooke et al. 1997) potentially by modulating the balance of protective and inflammatory chemokine/cytokines (Corasaniti et al. 2005; Kipp and Beyer 2009; Dhandapani et al. 2005; Bruce-Keller et al. 2007), or reducing oxidative changes in mitochondria (Simpkins et al. 2010). A cautionary note for all HIV studies is the potential for sex steroids, including progestins, to modulate infective processes (Asin et al. 2008; Zhang et al. 2008; Hel et al. 2010; Lee et al. 1997), which may complicate comparisons between epidemiological and experimental studies.

In conclusion, the data support the hypothesis that chronic exposure to HIV-1 Tat may play a significant role in the decline of cognitive and motor function in HIV patients, perhaps related to or driven by changes in specific cell populations. This study has examined cellular changes specifically within the striatum, but the behavioral deficits observed almost certainly will involve pathology in other CNS regions. Sex appears to be an important variable in predicting vulnerability to behavioral deficits, with males being significantly more affected in several standard rodent tests of motor and social/cognitive skills. Males also showed an enhanced state of glial activation and more evidence of synaptic damage, both of which have been related to cognitive and motor impairments in HIV patients. Importantly, Tat will be produced and released by residually infected CNS cells even when new infection is limited by cART (Johnson et al. 2013), suggesting its importance in determining the stability of CNS cell populations and motor and cognitive health during chronic infection.

## Electronic supplementary material

Below is the link to the electronic supplementary material.

Supplementary material 1 (DOCX 28 kb)
