# Supplementary material for: Effects of chronic HIV-1 Tat exposure in the CNS: heightened vulnerability of males versus females to changes in cell numbers, synaptic integrity, and behavior
Source: Brain Struct Funct. 2013 Dec 19;220(2):605–23. doi: 10.1007/s00429-013-0676-6 (PMC4341022; doi:10.1007/s00429-013-0676-6)
Supplement: Supplementary file 1 — Supplementary material 1 (DOCX 28 kb) [file 429_2013_676_MOESM1_ESM.docx]

**SUPPLEMENTARY DATA**

**Table 1**  Measurements of activity in open field and light/dark box tests

| A. |  |  |
| --- | --- | --- |
| Open field test | Travel distance (cm/10min ± SE) | |
|  | 6 month old | 6 month old |
|  | No DOX | 3 month DOX |
| Female Tat^-^ | 1904.68±334.36 | 2184.96±183.83 |
| Female Tat^+^ | 2484.53±116.25 | 1682.55±190.38 |
| Male Tat**^-^** | 2045.54±165.53 | 2027.74±128.80 |
| Male Tat^+^ | 2152.74±199.09 | 1689.94± 94.54 |
| B. |  |  |
| Light/Dark test | % of Travel distance in dark side | |
|  | (cm/10min ± SE) | |
|  | 6 month old | 6 month old |
|  | No DOX | 3 month DOX |
| Female Tat^-^ | 79.77 ± 12.03 | 80.28 ± 5.63 |
| Female Tat^+^ | 77.49 ± 7.57 | 83.44 ± 6.48 |
| Male Tat**^-^** | 71.71 ± 8.68 | 77.82 ± 3.01 |
| Male Tat^+^ | 71.39 ± 4.06 | 81.26 ± 4.69 |
